# Assessment of health-related quality of life in Australian patients with idiopathic pulmonary fibrosis: a comparison of the EQ-5D-5L and the AQoL-8D

**DOI:** 10.1007/s11136-022-03205-z

**Published:** 2022-08-04

**Authors:** Ingrid A. Cox, Julie Campbell, Barbara de Graaff, Petr Otahal, Tamera J. Corte, Yuben Moodley, Peter Hopkins, Sacha Macansh, E. Haydn Walters, Andrew J. Palmer

**Affiliations:** 1 Menzies Institute for Medical Research, University of Tasmania, 17 Liverpool Street, Hobart, TAS Australia; 2 NHMRC Centre of Research Excellence for Pulmonary Fibrosis, Melbourne, Australia; 3 Central Clinical School, The University of Sydney, Camperdown, Australia; 4 Department of Respiratory and Sleep Medicine, Royal Prince Alfred Hospital, Camperdown, Australia; 5 Faculty of Health and Medical Sciences, The University of Western Australia, Perth, Australia; 6 Institute of Respiratory Health, The University of Western Australia, Perth, Australia; 7 Department of Respiratory Medicine, Fiona Stanley Hospital, Murdoch, Australia; 8 Queensland Centre for Pulmonary Transplantation and Vascular Disease, The Prince Charles Hospital, Chermside, Australia; 9 Faculty of Medicine, University of Queensland, Brisbane, QLD Australia; 10 Australian Idiopathic Pulmonary Fibrosis Registry, Lung Foundation of Australia, New South Wales, Australia

**Keywords:** AQoL-8D, EQ-5D-5L, Utility values, Health-related quality of life, Idiopathic pulmonary fibrosis

## Abstract

**Purpose:**

Idiopathic pulmonary fibrosis (IPF) is a progressive and debilitating chronic lung disease with a high symptom burden, which has a substantial impact on health-related quality of life (HRQoL). Our study aimed to assess the suitability of the EuroQol five-dimension (EQ-5D-5L) and the Assessment of Quality of Life- eight-dimension (AQoL-8D) questionnaires in measuring HRQoL as health state utility values (HSUVs) in an Australian IPF cohort.

**Methods:**

Data for estimation of health state utility values (HSUVs) were collected from participants of the Australian IPF Registry (AIPFR) using self-administered surveys which included the EQ-5D-5L and the AQoL-8D. Data on lung function and disease specific HRQoL instruments were collected from the AIPFR. Performance of the two instruments was evaluated based on questionnaire practicality, agreement between the two instruments and test performance (internal and construct validity).

**Results:**

Overall completion rates for the EQ-5D-5L and AQoL-8D were 96% and 85%, respectively. Mean (median) HSUVs were 0.65 (0.70) and 0.69 (0.72) for the EQ-5D-5L and AQoL-8D, respectively. There was reasonable agreement between the two instruments based on the Bland–Altman plot mean difference (−0.04) and intraclass correlation coefficient (0.84), however there were some fundamental differences. A larger range of values was observed with the EQ-5D-5L (−0.57–1.00 vs 0.16–1.00). The EQ-5D-5L had a greater divergent sensitivity and efficacy in relation to assessing HSUVs between clinical groupings. The AQoL-8D ,however, had a higher sensitivity to measure psychosocial aspects of HRQoL in IPF.

**Conclusion:**

The EQ-5D-5L demonstrated superior performance when compared to AQoL-8D in persons with IPF. This may be attributable to the high symptom burden which is physically debilitating to which the EQ-5D-5L may be more sensitive.

**Supplementary Information:**

The online version contains supplementary material available at 10.1007/s11136-022-03205-z.

## Plain English summary

Idiopathic Pulmonary Fibrosis (IPF) is a progressive and fatal lung disease with a high symptom burden, which has a considerable impact on health-related quality of life (HRQoL). Numerous questionnaires have been developed for the purpose of evaluating HRQoL and deriving health state utility values (HSUV) which represents the preference of an individual for a particular health state. Each questionnaire may however produce different results in the same individual and this overall difference in values are primarily as a result of the descriptive systems. Consequently, it is important to understand these differences in the descriptive systems in choosing the appropriate questionnaire for economic evaluation. Our study aimed to compare the EQ-5D-5L and AQoL-8D to ascertain their performance to derive HSUVs in an Australian cohort of persons living with IPF.

Our results demonstrated that there was reasonable agreement between the two instruments with mean HSUVs for the EQ-5D-5L and AQoL-8D of 0.65 and 0.69 respectively. There were however some fundamental differences which lead us to conclude that the EQ-5D-5L demonstrated superior performance when compared to the AQoL-8D. This may be attributable to the high symptom burden associated with IPF and the inherent sensitivity of the EQ-5D-5L to measure physical attributes of HRQoL.

## Introduction

Idiopathic pulmonary fibrosis (IPF) is the most frequent type of interstitial lung disease in older adults, characterised by progressive fibrosis and scarring of lung tissue, invariably leading to declining lung function, respiratory failure, and death [1–3]. Considering the natural progression of the disease, IPF is associated with a high symptom burden, typified by chronic cough and progressive shortness of breath, both which have a huge impact on health-related quality of life (HRQoL) [3].

HRQoL is an important aspect in health economic assessments of interventions to manage IPF. It has become increasingly important given the expanding landscape of research for IPF therapies, especially considering the high costs associated with treatments and the heterogeneity of clinical outcomes that may be masked by the adverse effects of the therapies under assessment. A diverse number of patient reported outcome measures (PROM) have been used to quantify HRQoL in persons with IPF [4]. While there is no gold standard to measure HRQoL in persons with IPF, it is important to ensure that the instrument being used is sensitive enough to quantify changes in health status related to the intervention under investigation [5]. Many disease specific instruments are currently being used for IPF none of these are preference based [4]. Preference-based PROMs and in particular, multi-attribute utility instruments (MAUIs) are recommended for economic evaluations as they generate heath-state utility values (HSUVs). HSUVs are an important metric that are used to estimate quality adjusted life years (QALYs) [5]. Numerous MAUIs have been developed for this purpose. To derive HSUVs, these instruments make use of two components, a descriptive system which includes questions that describe a person’s health and a utility algorithm which translates the question responses into a value (HSUV) measured on a scale of 0.00 (death) to 1.00 (best health) but can also be negative which represents health states considered worse than death [6]. A recent review of national health technology assessment guidelines in several countries demonstrating that a few MAUIs dominate: the EuroQol 5 dimension suite of instruments (EQ-5D): 85%; Short Form-6 Dimension (SF-6D): 32%; the Health Utilities Index (HUI): 29%; Quality of Wellbeing (QWB): 9%; and Assessment of Quality of Life (AQoL): 6% [7]. Each MAUI may, however produce different HSUVs in the same individual primarily as a result of the descriptive systems [6]. Thus, it is important to understand differences in the descriptive systems when choosing the appropriate MAUI for health economic evaluations. Although the EQ-5D suite of instruments is cited as the most used and most recommended or preferred by health funding agencies, recent studies have demonstrated that it may not necessarily be the most suitable in all disease conditions [7, 8]. There are currently just a few studies that have utilised MAUIs to assess HRQoL in individuals with IPF, and in those that have, most used the EQ-5D suite of instruments [4]. The AQoL-8D instrument, most recently developed with the aim of addressing deficiencies in descriptive systems of existing MAUIs and is often used in the Australian context, however it has not been assessed for suitability in the context of IPF [9]. No studies have undertaken a comparison of MAUIs to assess their relative performance and influence of the descriptive systems in the context of IPF.

The aim of this study was to assess the performance of between the EQ-5D-5L and the AQoL-8D to measure HSUVs in an Australian cohort of persons living with IPF. More specifically, we aimed to do this by conducting a head-to-head comparison of the two MAUIs, taking into consideration the practicality of the questionnaires, the level of agreement and test performance, namely the internal and construct validity.

## Methods

### Study participants and data collection

Participants for this study were recruited between August 2018 and December 2019 from Australian IPF Registry (AIPFR) [10, 11]. The AIPFR is a national multi-centre, prospective registry of IPF patients facilitated by the Lung Foundation of Australia. Details on the recruitment methodology for the AIFPR have been previosly described and can also be found in the supplement [10, 11]. Participation was voluntary through informed consent, and withdrawal was possible at any time without reason.

Data were collected using a predesigned survey instrument. The instrument collected socio-demographic and clinical information and incorporated the EQ-5D-5L and AQoL-8D. Data for St. George’s Respiratory Questionnaire (SGRQ), Hospital Anxiety and Depression Scale (HADS), University of California San Diego Shortness of Breath Questionnaire (SOBQ) and pulmonary function tests (PFT) were collected from the AIPFR database, using those with the date of completion closest to the survey completion, but only those within 12 months. For purposes of comparison, demographic and clinical data on non-responders to the survey were also collected from the AIPFR database.

### Health-related quality of life measures

Table 1 provides a summary of the characteristics of the MAUIs, and disease specific instruments used in this study.

**Table 1:** Characteristics of the health-related quality of life instruments used in this study

	Preference based measures of HRQoL (MAUIs)		Disease specific or symptom related measures of HRQoL		
	EQ-5D-5L [5, 10, 47]	AQoL-8D [13, 14, 48]	SGRQ [16, 17]	SOBQ [18]	HADS [18]
Dimensions	5	8	3	NA	2
	Mobility	Mental health	Activity		Depression
	Pain/discomfort	Coping	Symptoms		Anxiety
	Depression/anxiety	Happiness	Impact (psychosocial)		
	Self-care	Relationships			
	Usual activities	Self-worth			
		Pain			
		Independent living			
		Senses			
	*Visual analogue scale:*	*Super dimensions:*	*Total score:*		
	Rates health on a scale of 0–100 (worst to best)	Psychosocial health (pain, independent living, senses)	Summary of all dimensions		
		Physical health (mental health, coping, happiness, relationships, self-worth)			
Items	5	35	50	24	14
Response levels/types	5	4–6	3–6 or True/false	6	4
Health states	3125	2.4 × 10^23^	NA	NA	NA
Measurement unit	Health state utility values (HSUVs)	Health state utility values (HSUVs)	Score	Score	Score
Estimation of score	Scoring algorithm	Scoring algorithm	Scoring algorithm	Total score	Total score
Scale	0–1.00	0–1.00	0–100%	0–120	0–7 Normal
	0 worst score/death	0 worst score/death	0 best possible health	0 no shortness of breath	8–10 borderline normal
	1 best health	1 best health	100 worst possible health	120 worst possible shortness of breath	11–21- anxiety/depression
	Scores less than 0 indicate health states worse than death	Scores less than 0 indicate health states worse than death			
Theoretical range	− 0.59–1.00	0.06–1.00	0–100	0–120	0–21
Validated for IPF	No	No	Yes	Yes	No
Missing values	Not allowed	Allows one missing value for dimensions with three to four items, two missing values for dimensions with seven to eight items	Allows two missed items for symptoms domain, four missed items for activity domain and six items for impact domain	Yes	Yes

*HRQoL* health-related quality of life, *HSUV* Health state utility value, *MAUI* multi-attribute utility instrument, *IPF* idiopathic pulmonary fibrosis, *EQ-5D-5L* EuroQol five-dimension- five level, *AQoL-8D* Assessment of quality of life eight-dimension *SGRQ, St.* George’s respiratory questionnaire, *SOBQ* University of California San Diego shortness of breath questionnaire, *HADS* Hospital depression and anxiety scale, *NA* not applicable

#### MAUIs

##### EQ-5D-5L

The EQ-5D-5L was developed to address the limited sensitivity of its predecessor the EQ-5D-3L [12]. In addition to generating HSUVs, the EQ-5D-5L also includes a visual analogue scale (EQ-VAS) which patients can use to rate their current health on a scale from 0 to 100 (worst to best) [12]. While the valuation process for the EQ-5D-5L has been completed in Australia, it is yet to be published [11, 13]. To estimate HSUVs for the EQ-5D-5L, we made use of an earlier study which developed utility weights for the EQ-5D-5L for Australia [11, 13]. To ensure the robustness of the HSUVs estimated, we conducted a sensitivity analysis using estimates generated using the crosswalk method by Van Huot et al. [14] and using the United Kingdom (UK) value set for EQ-5D-5L [15].

##### AQoL-8D

The AQoL-8D is the latest version of the AQoL suite of instruments. This MAUI was developed to improve the instrument’s sensitivity to capture and assess the psychosocial domains of HRQoL [9, 16]. AQoL-8D HSUVs were calculated using a scoring algorithm incorporating Australian weights [17].

#### Comparator HRQoL instruments

Given that the EQ-5D-5L and AQoL-8D are preference-based instruments, we compared these with non-preference based instruments HRQoL measures used for IPF patients, namely disease specific instruments such as the SGRQ [18, 19], SOBQ [20] and others such as the HADS [21]. Scores for the SGRQ and SOBQ were presented as quartiles.

### Disease severity

Several disease severity classification systems have been used for IPF [22, 23]. We used three measures: (1) the Gender, Age, Physiology (GAP) staging [24]; (2) the Composite Physiological Index (CPI) [25]; and (3) the forced vital capacity as a percent predicted (FVC%) [26]. These are fully described in the supplement.

### Medications

Treatments were categorised in accordance with international guidelines for IPF, classified as (1) conditional recommendation for use (anti-fibrotics pirfenidone and nintedanib); (2) conditional recommendation for use (limited evidence n-acetylcysteine and anti-reflux medications); and (3) strong recommendations against use (prednisolone, warfarin, and azathioprine) [27, 28].

### Statistical analysis

#### Descriptive statistics

Statistical analyses were conducted using R Software and STATA statistical software [29, 30]. Participants for whom a HSUV could be generated for one or both instruments (AQoL-8D or EQ-5D-5L) were included in this analysis. Two sample *t*-test or Chi-squared tests were used where appropriate to compare (1) responders and non-responders to the survey (2) participants with PFTs and participants without/with incomplete PFTs and (3) participants with comparator HRQoL data and participants without. A *p*-value < 0.05 was used as a test for statistical significance. Characteristics of participants are presented descriptively as means and standard deviations (SD), medians and interquartile range (IQR) for continuous variables or counts and proportions for categorical variables.

Summary statistics for participants’ characteristics and HSUV scores for the EQ-5D-5L, AQoL-8D, and the EQ-VAS were summarised as means and 95% confidence intervals (95%CI) and medians (IQR). Ceiling and floor effects for both instruments were evaluated by calculating the proportion of persons in the best possible and worst health states, described as 1.00 and ≤ 0.00, respectively. Response levels for all dimensions of EQ-5D-5L and AQoL-8D were evaluated and ratings for each level of each dimension were analysed.

#### Questionnaire practicality

Given the debilitating nature of IPF, an important criterion for evaluation is the practicality of the questionnaire. Firstly, we evaluated the completion rate of the questionnaire by assessing the number of complete questionnaires and number of questionnaires with sufficient information for utility calculation. Secondly, noting the disabling symptoms associated with the disease, we reviewed whether there were questions in both instruments where extreme (severe) responses were not recorded as expected, which would provide an indication of the meticulousness of responses under symptom duress.

#### Agreement between instruments

Pairwise agreement between the HSUVs generated by each instrument for individual participants was first assessed using a scatterplot. Bland Altman plots were then used to assess agreement between the two instruments by plotting the differences between the HSUVs of the two instruments against the mean of the two HSUVs along with the 95% confidence limits of agreement [31]. Intraclass correlation coefficients (ICCs) were then calculated using a two-way random effects model with average measures and absolute agreement in accordance with the nonparametric nature of the data [32]. An ICC < 0.50 is indicative of poor agreement; 0.50–0.75 moderate; 0.75–0.9 good; and > 0.90 excellent agreement [33]. We also evaluated scores across all instruments and disease severity measures for participants who demonstrated floor and ceiling effects [34]. Lastly, we evaluated the influence of sociodemographic and clinical covariates on HSUVs using Tobit models [35].

#### Test performance

##### Internal validity

Internal validity was assessed using the Cronbach’s alpha. For items within each dimension of the AQoL-8D, values > 0.7 were considered as acceptable levels of reliability [36].

##### Construct validity

To assess convergent validity, we assessed the strength of correlation between the two MAUIs and additionally between the MAUIs and other measures of HRQOL using Spearman’s rank correlation coefficient [37]. A Spearman’s rho ≥ 0.8 or ≤ −0.8 was considered a very strong association; 0.60–0.79 or −0.60 to 0.79 a strong association; 0.40–0.59 or −0.40 to 0.59 a moderate association; and −0.40 to 0.40 a weak association [38].

To assess divergent validity, we evaluated known group validity and the ability of the instruments to detect clinically relevant differences, more specifically in relation to the FVC%, GAP and CPI. For known group validity we utilised the Kruskal–Wallis rank test to assess the differences within clinical variable groups [37]. To assess the ability of the instruments to detect clinically relevant differences we estimated the effect size (ES), relative efficiency (RE) with the EQ-5D-5L as the reference, and the area under receiver operating characteristics curves (AUC) [37]. RE values > 1 would indicate the AQoL-8D is more efficient in distinguishing between known groups and clinical levels [37].

## Results

### Participants’ characteristics

Table 2 and S1 provide a summary of participant and non-participant characteristics. There was a 56% response rate (Figure S1). Of the 162 respondents, 156 completed the EQ-5D-5L and 157 the AQoL-8D. Persons who did not participate in the study (*n* = 126) had more comorbidities and were older than responders. Participants with lung function (*n* = 105) and comparator HRQoL data (*n* = 129) were more likely to be on antifibrotic medication (Table S1).

**Table 2:** Participant characteristics

	Participant characteristic (*n* = 162)
*Age*	
Mean (SD)	73.8 (7.6)
Median [IQR]	74.0 [69–78]
*Age group, n (%)*	
< 65	19 (11.7)
65–75	82 (50.6)
75–85	48 (29.6)
> 85	13 (8.0)
*Gender, n (%)*	
Male	99 (61.1)
Female	63 (38.9)
*Race, n (%)*	
Caucasian	145 (89.5)
Other	9 (5.6)
Missing	8 (4.9)
*Jurisdiction, n (%)*	
NSW	66 (40.7)
VIC	31 (19.1)
QLD	14 (8.6)
SA	25 (15.4)
TAS	16 (9.9)
WA	6 (3.7)
ACT	2 (1.2)
NT	2 (1.2)
*Remoteness area, n (%)*	
Major city	99 (61.1)
Inner regional	43 (26.5)
Outer regional	15 (9.3)
Remote	1 (0.6)
Missing	4 (2.5)
*Marital Status, n (%)*	
Married/De facto/Partner	115 (71.0)
Divorced/Widowed/Separated/Single	45 (27.8)
Missing	2 (1.2)
*Employment, n (%)*	
Full time/Part time/Unpaid work	19 (11.7)
Retired	135 (83.3)
Unemployed	7 (4.3)
Missing	1 (0.6)
*Income ($AUD), n (%)*	
< 400/week	56 (35.5)
400–799/week	50 (29.0)
800–1249/week	15 (9.0)
> 1250/week	12 (7.7)
Missing	29 (18.7)
*Comorbidities n (%)*	
0	33 (20.4)
1	56 (34.6)
2	41 (25.3)
> 2	32 (19.8)
*BMI kg/m2 (n* = *153)*	
Mean ± SD	28.1 (4.8)
Median [IQR]	27.6[24.9–31.1]
*FVC%, (n* = *101)*	
Mean (SD)	87.6 (22.3)
Median [IQR]	85.0[73.1- 99.7]
*GAP total, (n* = *97)*	
Mean (SD)	4 (1)
Median [IQR]	4 (3–5)
*CPI total, (n* = *97)*	
Mean (SD)	36.0 (13.8)
Median [IQR]	45.5[34.2–54.9)
*Drugs, n (%)*	
Conditional recommendations for use (antifibrotics)	96 (59.2)
Conditional recommendations for use (limited evidence)	81 (50.0)
Strong recommendations against use	27 (16.7)
*St George’s Respiratory Questionnaire Total Score, (n* = *122)*	
Mean (SD)	46.0 (20.6)
Median[IQR]	44.9 [31.5,63.9]
*UCSD Shortness of Breath Questionnaire, (n* = *122)*	
Mean (SD)	40.2 (27.6)
Median[IQR]	33.5 [16.3,62.0]
*Hospital anxiety and depression questionnaire, (n* = *122)*	
Anxiety	
Mean (SD)	4.8 (4.1)
Median (IQR)	4 (1–7)
Depression	
Mean (SD)	4 (3)
Median (IQR)	4 (2–6)

Conditional recommendations for use (antifibrotics) drugs include pirfenidone and nintedanib. Strong recommendations against use drugs include prednisolone, n-acetylcysteine, warfarin, and azathioprine. Conditional recommendations for use (limited evidence) includes anti-reflux drugs

*n* number of participants, *SD* Standard deviation, *FVC* forced vital capacity percent predicted, *CPI* Composite physiologic index, *GAP* Gender age, physiology index, *BMI* Body Mass Index, *IQR* interquartile range, *UCSD* University of California, San Diego, *NSW* New South Wales, *VIC* Victoria, *SA* South Australia, *QLD* Queensland, *TAS* Tasmania, *WA* Western Australia, *ACT* Australian Capital Territory, *NT* Northern Territory

The mean age for participants was 73.8 (7.6) years and 80% were aged 65–85 years. Most participants were male (61%), Caucasian (90%), lived in major cities (61%) and were from New South Wales (41%). Three-fifths were on antifibrotic treatment (60%) and 80% had ≥ 1 comorbidity.

The mean GAP index, FVC % and CPI were 4 (1), 87.6 (22.4) and 36.0 (13.8) respectively. Mean scores for total SGRQ and SOBQ were 46.0 (20.6) and 40.2 (27.6) respectively. The HADS questionnaire detected depression and anxiety in 24% and 16% of participants, respectively.

### Questionnaire practicality

#### Completion of the questionnaires

Of the 162 participants, 97% completed the AQoL-8D with sufficient data for utility derivation but only 85% fully completed the questionnaire. For the EQ-5D-5L, 96% completed the questionnaire.

#### Item responses

Less than 1% of participants had severe problems with pain/discomfort (PD) and self-care (SC) (Table S2). For PD, 67% of participants had slight/no pain and for self-care, 86%. For mobility and anxiety or depression (AD), 1% had severe problems while 87% of participants reported slight or no problems for AD and 62% for mobility. For usual activities (UA), 4% had severe problems and 66% reported slight or no problems.

For the AQoL-8D, < 2% of participants had severe issues with mental health, happiness, relationships, self-worth, and senses. For pain and coping, responses for the severe level were 4% and 6% respectively, and 64–77% rated themselves as having slight or no deficit/problems in all dimensions.

### Agreement between instruments

**Fig. 1:**
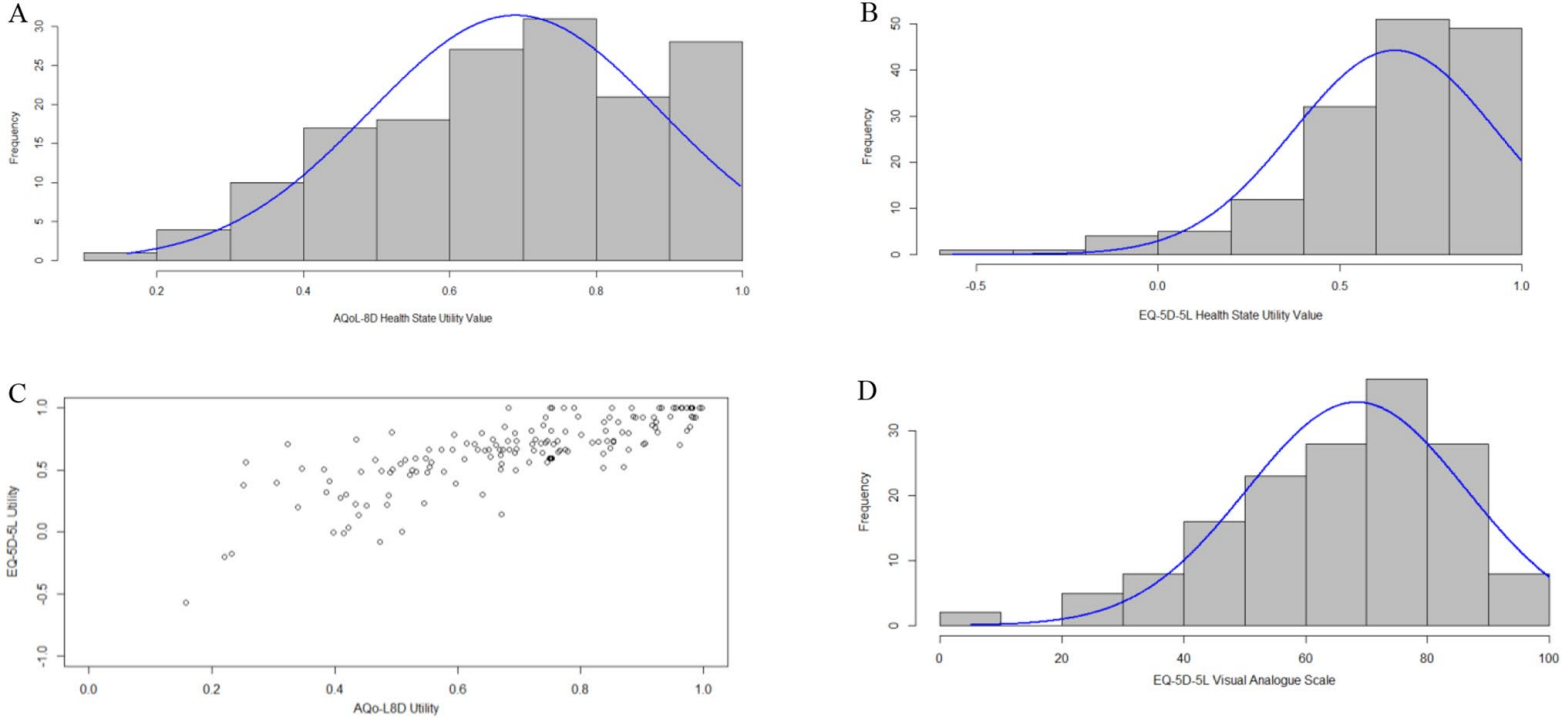
Distribution of scores for AQoL-8D, EQ-5D-5L and EQ-VAS

**Fig. 2:**
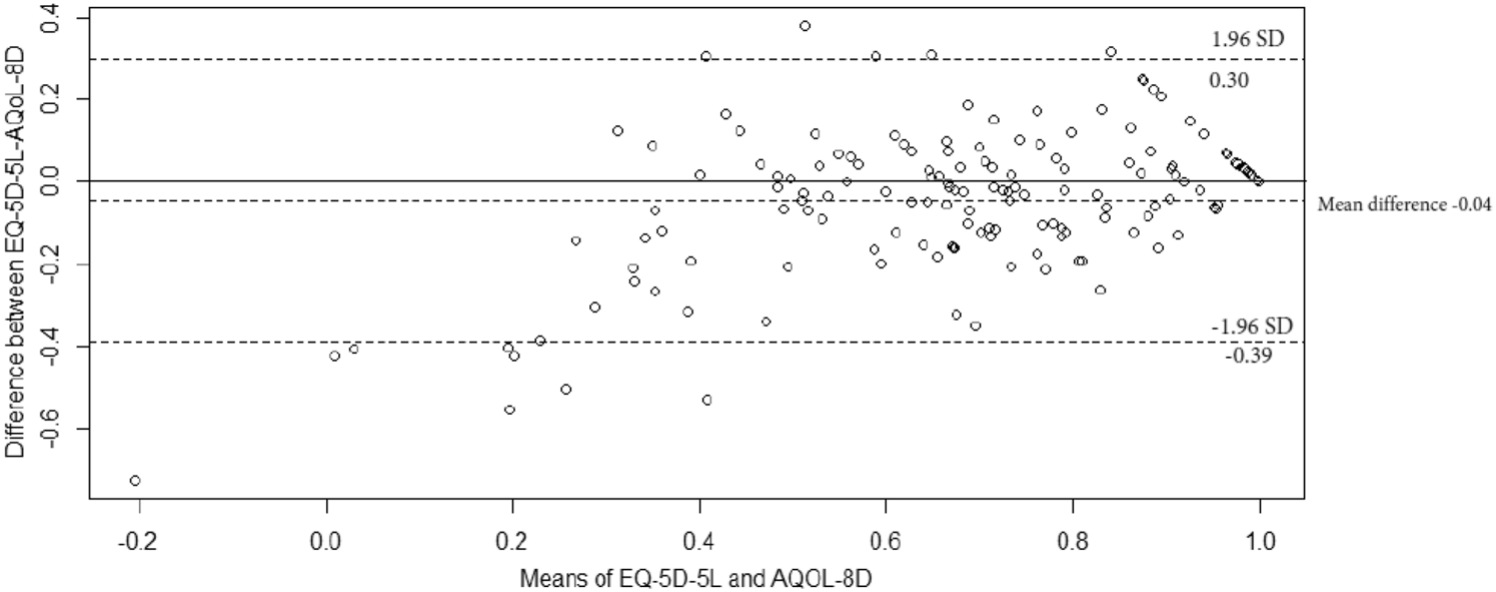
Bland Altman plot for differences in means for AQoL-8D and EQ-5D-5L utilities

Figure 1A and 1B show distribution of HSUVs for the EQ-5D-5L and AQoL-8D, both of which were left-skewed. Table 3 provides summary statistics for the instruments. The EQ-5D-5L exhibited a wider range of values (−0.57 to 1.00) with 4% of participants (*n* = 6) reporting scores less than 0 (floor effect) and 13% (*n* = 20) the ceiling effect. The AQoL-8D scores ranged between 0.16 and 1.00 with only 1% (*n* = 2) demonstrating a ceiling effect. Mean (SD) for the EQ-5D-5L, AQoL-8D and EQ-VAS were 0.65(0.28), 0.69(0.20) and 69 (18), respectively. The scatterplot for the two instruments (Fig. 1C) showed clustering in the upper right quadrant corresponding to HSUVs higher than 0.50 for the EQ-5D and higher than 0.70 for the AQoL. The agreement between the two instruments was good with an ICC of 0.84 (95%CI, 0.78–0.89). The Bland Altman plot (Fig. 2) demonstrated a similar trend with a negative mean difference (−0.04) between the two instruments, with 92.1% of the HSUVs between the bounds of agreement (−0.39 to 0.30).

**Table 3:** Summary statistics for AQoL-8D, EQ-5D-5L, and EQ-VAS

Instrument	AQoL 8D	EQ-5D-5L	EQ-5D VAS
*N*	157	155	156
Mean (SD)	0.69 (0.20)	0.65 (0.28)	69 (18)
Median (IQR)	0.72 (0.55–0.85)	0.70 (0.52–0.84)	70 (60–80)
Skewness	− 0.41	− 1.27	− 0.84
Observed range	0.16–1.00	− 0.57 to 1.00	5–100
Theoretical range [47, 48]	0.06–1.00	− 0.59 to 1.00	0–100
Participants on floor, *n* (%)	0 (0)	6 (4)	0 (0)
Participants on ceiling, *n* (%)	2 (1)	20 (13)	2 (1)

*n* number of participants, *SD* Standard deviation, *IQR* interquartile range

Tables 3 and 4 provide a comparison of participants with ceiling and floor effects from the EQ-5D-5L to the EQ-VAS, AQoL-8D, disease specific HRQoL instruments and disease severity measures. Of the 20 participants reporting perfect health, almost all (*n* = 18) had lower AQoL-8D scores driven by the MSD, which ranged between 0.33 and 0.87 with a mean of 0.41 (0.21). Overall, there were varying levels of concordance between lung function variables and the HRQoL measures. Similar trends were noted for the participants with floor effects. The participant with the lowest EQ-5D-5L utility (−0.57) did not have corresponding low lung function measures, however, they did record the worst scores for the SGRQ total (84), activity (100), and symptoms domain (97) and poor scores for the impact domain (70). The poor SGRQ impact domain score corresponded with the low AQoL MSD score (0.05) and poor HADS depression (12) and anxiety (16) scores which indicated moderate to severe anxiety and depression. While this participant recorded a low EQ-VAS score (32), it was not the lowest score recorded.

**Table 4:** Comparison of participants with perfect health based on the EQ-5D-5L

Participant	FVC% quintiles	GAP stage	CPI quintiles	EQ-VAS Score	AQOL-8D Utility	AQOL-8D MSD	AQOL-8D PSD	SGRQ total	SGRQ symptoms	SGRQ activity	SGRQ impact	SOBQ	HADS anxiety	HADS depression
1	2	-	-	80	0.68	0.36	0.59	25	24	32	22	22	1	4
2	5	1	1	90	0.75	0.33	0.89	15	24	0	22	22	4	6
3	4	1	1	85	0.75	0.33	0.90	28	26	47	18	18	2	2
4	3	2	5	90	0.77	0.38	0.79	39	41	47	33	33	6	7
5	-	-	-	70	0.79	0.38	0.87	50	63	49	47	47	2	2
6	4	2	2	80	0.85	0.52	0.71	34	36	47	24	24	0	3
7	1	2	3	70	0.88	0.52	0.92	62	100	66	48	48	6	5
8	5	-		88	0.93	0.60	0.88	70	76	72	66	66	4	1
9	1	2	2	85	0.93	0.66	0.84	17	19	29	9	9	1	1
10	3	1	5	90	0.95	0.67	0.91	22	41	29	12	12	1	2
11	5	2	1	85	0.95	0.65	1.00	37	57	66	14	14	5	2
12	3	1	2	80	0.96	0.78	0.85	14	21	17	10	10	0	1
13	4	1	1	95	0.97	0.75	0.91	12	38	23	0	0	0	0
14	5	1	1	-	0.97	0.75	0.95	8	27	12	0	0	0	3
15	1	2	3	85	0.98	0.78	0.97	25	48	23	21	21	0	1
16	1	2	4	93	0.98	0.74	1.00	22	32	35	11	11	1	3
17	4	1	2	95	0.98	0.83	0.88	29	41	59	9	9	1	0
18	4	2	2	95	0.99	0.87	0.95	14	6	24	11	11	0	0
19	3	1	1	100	1.00	0.93	0.95	0	0	0	0	0	0	0
20	5	1	2	96	1.00	0.92	0.95	4	13	0	4	4	0	0
Mean (SD)	–	–	–	69 (78)	0.69 (0.20)	0.41 (0.21)	0.60 (0.20)	45 (20)	50 (24)	62 (23)	22 (34)	39 (28)	5 (4)	4 (3)
Median (IQR)	–	–	–	70 (60–80)	0.72 (0.55–0.85)	0.37 (0.26–0.52)	0.59 (0.46–0.76)	42 (31–60)	50 (32–71)	66 (48–79)	30 (17–49)	32 (16–62)	4 (1–7)	3 (2–6)

*SD* standard deviation, *IQR* interquartile range, *FVC%* forced vital capacity percent predicted, *GAP* gender, age and physiology, *CPI* composite physiological index, *MSD* mental health super-dimension, *PSD* physical super-dimension, *SGRQ* St George’s Respiratory Questionnaire, *SOBQ* University of California San Diego shortness of breath questionnaire, *HADS* Hospital Anxiety and Depression Scale, *SGRQ* scores from 0 to 100, 100 being worst score. Impact domain represents social and psychosocial impact. UCSD SOB: 0–120, 120 being worst score. HADS, 0–7: normal, 8–10: borderline abnormal, 11–21: abnormal. GAP stage 1: mild; GAP stage 2: moderate; GAP stage 3, severe

Table 5 provides summary statistics for AQoL-8D and EQ-5D-5L HSUVs and EQ-VAS by participant characteristics. Males generally had higher HSUVs as measured by both instruments and the EQ-VAS. While there was no distinct trend observed for mean HSUVs by age group, persons in the youngest age group (≤ 65 years) had the lowest HSUVs for both instruments and the EQ-VAS. There was an overall reduction in mean HSUVs with increasing disease severity for both instruments and the EQ-VAS as demonstrated by the FVC%, GAP stage and CPI score. Participants with better scores on the SGRQ, SOBQ, and HADS had higher HSUVs and EQ-VAS scores. HSUVs and EQ-VAS scores decreased with increasing number of comorbidities. Participants who were on antifibrotic medication consistently had higher HSUVs for both instruments and on the EQ-VAS compared to those not receiving antifibrotics. Conversely, persons who were on medication categories “conditional recommendation for use” and “strong recommendations against use” had lower HSUVs than those who were not on these medications for both instruments and the EQ-VAS. Employed participants had higher HSUVs and EQ-VAS scores than unemployed and retired participants.

**Table 5:** Comparison of participants with the floor effect on the EQ-5D-5L

Participant	EQ-5D-5L Utility	FVC% quintiles	GAP stage	CPI quintiles	EQ-VAS Score	AQOL-8D Utility	AQOL-8D MSD	AQOL-8D PSD	SGRQ total	SGRQ symptoms	SGRQ activity	SGRQ impact	SOBQ	HADS anxiety	HADS depression
1	− 0.57	5	1	2	32	0.16	0.05	0.19	84	97	100	70	73	16	12
2	− 0.20	1			13	0.22	0.11	0.18	81	64	93	79	91	10	8
3	− 0.17	1	1	3	40	0.23	0.08	0.29	49	75	66	30	38	12	2
4	− 0.08	2	2	3	25	0.47	0.17	0.51	73	83	93	59	101	9	14
5	− 0.01	1	3	5	40	0.41	0.20	0.30	64	88	93	38	63	7	6
6	− 0.01	1	2	4	32	0.40	0.15	0.38	61	66	100	38	79	7	4
Mean (SD)	− 0.17 (0.19)	–	–	–	32 (13)	0.32 (0.12)	0.12 (0.05)	0.31 (0.11)	68 (12)	79 (12)	91 (11)	52 (18)	74 (20)	10 (3)	8 (4)
Median (IQR)	− 0.13 (− 0.2 to − 0.03)	–	–	–	40 (25–40)	0.31 (0.22–0.41)	0.13 (0.08–0.16)	0.3 (0.22–0.36)	68 (61–79)	79 (68–87)	93 (93–98)	48 (38–67)	76 (66–88)	10 (8–12)	7 (5–11)

*SD* standard deviation, *IQR* interquartile range, *FVC%* forced vital capacity percent predicted, *GAP* gender, age and physiology, *CPI* composite physiological index, *MSD* mental health super-dimension, *PSD* physical super-dimension, *SGRQ* St George’s Respiratory Questionnaire, *SOBQ* University of California San Diego shortness of breath questionnaire, *HADS* Hospital Anxiety and Depression Scale, *SGRQ* scores from 0 to 100, 100 being worst score. Impact domain represents social and psychosocial impact. UCSD SOB: 0–120, 120 being worst score. HADS, 0–7: normal, 8–10: borderline abnormal, 11–21: abnormal. GAP stage 1: mild; GAP stage 2: moderate; GAP stage 3: severe

**Table 6:** AQoL8D and EQ-5D-5L utility scores and EQ-5D Visual analogue scale scores stratified by participant characteristics

	EQ-VAS				EQ-5D-5L				AQoL-8D			
	*n* (%)	Mean HSUV (95CI)	Median (IQR)	Range (min–max)	*n* (%)	Mean HSUV (95CI)	Median (IQR)	Range (min–max)	*n* (%)	Mean HSUV (95CI)	Median (IQR)	Range (min–max)
*All participants*^*a*^	156 (100)	68 (66–71)	70 (60–80)	5–100	155 (100)	0.65 (0.61–0.70)	0.70 (0.52–0.84)	−0.57 to 1.00	157 (100)	0.69 (0.66–0.72)	0.72 (0.55–0.85)	0.15–1.00
*Age group (years)*												
≤ 65	17 (11)	58 (47–69)	60 (45–75)	25–100	17 (11)	0.56 (0.37–0.75)	0.62 (0.39–0.85)	−0.20 to 1.00	18 (11)	0.56 (0.44–0.68)	0.55 (0.39–0.69)	0.22–1.00
65–75	79 (50)	72 (68–76)	75 (63–85)	5–100	78 (50)	0.66 (0.60–0.73)	0.69 (0.52–0.91)	−0.57 to 1.00	79 (50)	0.70 (0.66–0.75)	0.72 (0.55–0.85)	0.16–1.00
75–85	47 (30)	68 (63–73)	70 (60–80)	10–95	47 (30)	0.64 (0.57–0.72)	0.67 (0.56–0.79)	−0.08 to 1.00	47 (30)	0.70 (0.65–0.76)	0.74 (0.57–0.85)	0.25–0.98
> 85	13 (8)	63 (52–74)	60 (50–75)	30–95	13 (8)	0.72 (0.61–0.82)	0.73 (0.71–0.79)	0.30–1.00	13 (8)	0.75 (0.67–0.83)	0.7 (0.64–0.85)	0.55–0.98
*Sex*												
Male	96 (61)	69 (65–73)	70 (60–85)	5–100	99 (64)	0.68 (0.63–0.73)	0.71 (0.57–0.87)	−0.57 to 1.00	95 (61)	0.71 (0.67–0.75)	0.74 (0.55–0.85)	0.16–1.00
Female	60 (38)	67 (63–71)	70 (59–80)	25–90	56 (36)	0.60 (0.52–0.68)	0.66 (0.49–0.80)	−0.20 to 1.00	62 (39)	0.66 (0.61–0.71)	0.68 (0.51–0.85)	0.22–0.98
Ethnicity*												
Caucasian	139 (89)	69 (66–72)	70 (60–80)	5–100	139 (90)	0.65 (0.60–0.69)	0.70 (0.51–0.84)	−0.57 to 1.00	141 (90)	0.69 (0.66–0.72)	0.72 (0.55–0.85)	0.16–1.00
Other	9 (6)	75 (64–86)	80 (60–85)	50–90	9 (6)	0.72 (0.59–0.84)	0.70 (0.58–0.81)	0.51–1.00	9 (6)	0.65 (0.46–0.83)	0.72 (0.47–0.76)	0.25–0.93
Employment status**												
Full time/Part time /Unpaid work	19 (12)	78 (70–86)	80 (70–88)	35–100	18 (12)	0.81 (0.73–0.81)	0.85 (0.67–0.92)	0.52–1.00	18 (11)	0.84 (0.78–0.91)	0.87 (0.75–0.95)	0.59–1.00
Retired	129 (82)	68 (64–71)	70 (60–80)	5–96	129 (83)	0.63 (0.59–0.63)	0.67 (0.51–0.81)	−0.57 to 1.00	131 (83)	0.67 (0.64–0.71)	0.69 (0.52–0.84)	0.16–1.00
Unemployed	7 (4)	57 (38–76)	60 (45–73)	25–80	7 (5)	0.53 (0.21–0.53)	0.66 (0.47–0.70)	−0.17 to 0.86	7 (4)	0.56 (0.36–0.75)	0.67 (0.41–0.72)	0.23–0.75
Income ($AUD)***												
< 400 /week	55 (35)	69 (65–73)	70 (60–80)	25–96	55 (35)	0.62 (0.54–0.69)	0.66 (0.51–0.79)	−0.57 to 1.00	55 (35)	0.67 (0.61–0.72)	0.71 (0.54–0.77)	0.16–1.00
400–799/week	47 (30)	67 (62–72)	70 (59–83)	25–95	45 (29)	0.67 (0.59–0.75)	0.70 (0.52–0.88)	−0.20 to 1.00	48 (31)	0.70 (0.64–0.76)	0.70 (0.55–0.89)	0.22–0.99
800–1249/week	14 (9)	70 (57–83)	75 (70–80)	10–95	14 (9)	0.63 (0.42–0.84)	0.72 (0.45–0.97)	−0.08 to 1.00	14 (9)	0.68 (0.57–0.8)	0.73 (0.49–0.83)	0.39–0.98
≥ 1250/week	12 (8)	72 (61–83)	70 (64–79)	35–100	12 (8)	0.76 (0.62–0.90)	0.81 (0.63–0.90)	0.21–1.00	12 (8)	0.74 (0.62–0.86)	0.76 (0.63–0.87)	0.45–1.00
Marital status****												
Married/de facto/partner	111 (71)	70 (67–73)	70 (60–80)	5–100	116 (75)	0.65 (0.60–0.70)	0.69 (0.52–0.85)	−0.57 to 1.00	113 (72)	0.70 (0.66–0.73)	0.74 (0.55–0.85)	0.16–1.00
Divorced/widowed/single/separated	43 (27)	65 (59–71)	65 (50–80)	25–88	37 (24)	0.66 (0.58–0.73)	0.70 (0.52–0.78)	−0.01 to 1.00	42 (27)	0.67 (0.61–0.74)	0.67 (0.55–0.85)	0.25–0.98
Remoteness Area*****												
Major city	97 (62)	69 (66–73)	70 (60–85)	5–100	95 (63)	0.68 (0.63–0.73)	0.70 (0.56–0.87)	−0.2 to 1.00	98 (62)	0.71 (0.67–0.75)	0.74 (0.56–0.88)	0.22–1.00
Outer regional	41 (26)	69 (64–75)	75 (60–80)	25–95	41 (27)	0.62 (0.52–0.73)	0.71 (0.51–0.85)	−0.57 to 1.00	41 (26)	0.66 (0.6–0.72)	0.68 (0.53–0.78)	0.16–0.98
Inner regional	13 (8)	58 (48–69)	60 (45–70)	25–85	15 (10)	0.58 (0.44–0.72)	0.63 (0.49–0.72)	−0.17 to 0.92	14 (9)	0.64 (0.52–0.75)	0.68 (0.54–0.77)	0.23–0.90
Remote	1 (1)	b	b	b	1 (1)	b	b	b	1 (1)	b	b	b
*Jurisdiction*												
NSW	63 (40)	71 (67–76)	75 (60–85)	10–100	62 (41)	0.68 (0.61–0.74)	0.71 (0.56–0.88)	−0.08 to 1.00	65 (41)	0.71 (0.66–0.75)	0.74 (0.56–0.87)	0.30–0.99
VIC	30 (19)	71 (65–77)	70 (60–85)	35–96	31 (20)	0.71 (0.61–0.80)	0.73 (0.59–0.22)	0.00–1.00	30 (19)	0.72 (0.64–0.8)	0.73 (0.55–0.91)	0.25–1.00
QLD	13 (8)	66 (57–75)	65 (55–80)	45–90	13 (9)	0.56 (0.36–0.77)	0.59 (0.30–0.81)	−0.20 to 1.00	13 (8)	0.61 (0.49–0.72)	0.67 (0.49–0.69)	0.22–0.96
SA	24 (15)	62 (54–70)	65 (54–75)	25–90	24 (16)	0.59 (0.45–0.72)	0.66 (0.51–0.73)	−0.57 to 1.00	24 (15)	0.66 (0.57–0.75)	0.73 (0.54–0.83)	0.16–0.98
TAS	16 (10)	61 (52–69)	63 (44–71)	40–90	16 (11)	0.58 (0.42–0.74)	0.65 (0.49–0.74)	−0.17 to 1.00	16 (10)	0.66 (0.55–0.77)	0.72 (0.66–0.78)	0.23–0.95
WA	6 (4)	68 (35–100)	83 (73–85)	5–85	5 (3)	0.70 (0.32–1.08)	0.70 (0.66–0.92)	0.22–1.00	6 (4)	0.71 (0.53–0.89)	0.87 (0.80–0.93)	0.43–0.96
ACT	2 (1)	b	60–100	60–100	2 (1)	b	b	0.64–1.00	2 (1)	†	†	0.74–1.00
NT	2 (1)	b	b	b	2 (1)	b	b	0.48–0.71	1 (1)	b	b	b
*FVC% quintiles*												
Quintile 1	20 (13)	59 (49–59)	59 (50–75)	10–93	20 (13)	0.48 (0.26–0.48)	0.61 (0.16–0.79)	−0.57 to 1.00	21 (13)	0.63 (0.52–0.74)	0.67 (0.47–0.80)	0.16–0.98
Quintile 2	17 (11)	68 (62–68)	70 (60–75)	50–90	18 (12)	0.67 (0.58–0.67)	0.69 (0.61–0.78)	0.14–0.93	18 (11)	0.70 (0.62–0.78)	0.68 (0.62–0.84)	0.38–0.95
Quintile 3	20 (13)	73 (64–73)	75 (60–85)	30–100	21 (14)	0.75 (0.67–0.75)	0.73 (0.58–0.88)	0.49–1.00	23 (15)	0.76 (0.67–0.85)	0.82 (0.59–0.94)	0.25–1.00
Quintile 4	20 (13)	79 (74–79)	83 (70–90)	55–95	21 (14)	0.78 (0.71–0.78)	0.79 (0.66–0.93)	0.56–1.00	19 (12)	0.78 (0.72–0.85)	0.75 (0.7–0.88)	0.55–0.98
Quintile 5	19 (13)	75 (67–75)	80 (68–85)	40–96	21 (14)	0.71 (0.57–0.71)	0.81 (0.59–0.92)	−0.17 to 1.00	21 (13)	0.72 (0.61–0.83)	0.75 (0.51–0.93)	0.23–1.00
NC	53 (36)	66 (60–66)	70 (55–80)	5–90	54 (36)	0.60 (0.53–0.60)	0.64 (0.48–0.73)	−0.01 to 1.00	55 (35)	0.63 (0.59–0.68)	0.67 (0.5–0.75)	0.25–0.96
*GAP*												
I	37 (24)	77 (72–82)	80 (70–90)	40–100	42 (28)	0.70 (0.61–0.79)	0.73 (0.58–0.92)	−0.20 to 1.00	42 (27)	0.71 (0.64–0.78)	0.74 (0.54–0.91)	0.22–1.00
II	46 (30)	69 (64–74)	70 (60–80)	10–95	46 (30)	0.72 (0.64–0.79)	0.74 (0.66–0.88)	−0.08 to 1.00	47 (30)	0.76 (0.71–0.81)	0.77 (0.65–0.88)	0.47–0.99
III	9 (6)	54 (40–68)	50 (49–70)	25–80	9 (6)	0.37 (0.04–0.70)	0.49 (0.20–0.62)	−0.57 to 0.78	9 (6)	0.54 (0.37–0.7)	0.52 (0.42–0.74)	0.16–0.80
NC	57 (38)	66 (61–71)	70 (55–80)	5–90	58 (38)	0.61 (0.54–0.67)	0.64 (0.49–0.73)	−0.01 to 1.00	59 (38)	0.64 (0.59–0.69)	0.68 (0.5–0.76)	0.25–0.96
*CPI Quintiles*												
Quintile 1	22 (14)	59 (49–68)	59 (49–75)	10–93	21 (14)	0.78 (0.7–0.85)	0.73 (0.66–0.92)	0.51–1.00	20 (13)	0.76 (0.67–0.85)	0.75 (0.64–0.93)	0.35–1.00
Quintile 2	17 (11)	66 (58–73)	65 (60–75)	30–90	18 (12)	0.75 (0.59–0.9)	0.83 (0.67–0.98)	−0.17 to 1.00	18 (11)	0.76 (0.64–0.88)	0.85 (0.58–0.98)	0.23–1.00
Quintile 3	22 (14)	73 (65–80)	75 (60–85)	30–100	18 (12)	0.67 (0.52–0.83)	0.76 (0.52–0.88)	−0.20 to 1.00	19 (12)	0.72 (0.63–0.82)	0.75 (0.65–0.85)	0.22–0.98
Quintile 4	19 (12)	79 (73–84)	80 (70–89)	55–95	19 (13)	0.66 (0.53–0.78)	0.72 (0.59–0.8)	−0.08 to 1.00	20 (13)	0.72 (0.63–0.81)	0.76 (0.58–0.88)	0.38–0.98
Quintile 5	20 (13)	73 (65–81)	80 (64–85)	40–96	21 (14)	0.54 (0.38–0.71)	0.66 (0.49–0.72)	−0.57 to 1.00	21 (13)	0.64 (0.55–0.72)	0.67 (0.52–0.77)	0.16–0.95
NC	56 (36)	66 (61–71)	70 (59–80)	5–90	58 (38)	0.61 (0.54–0.67)	0.64 (0.49–0.73)	−0.01 to 1.00	59 (38)	0.64 (0.59–0.69)	0.68 (0.5–0.76)	0.25–0.96
*Number of comorbidities*												
0	30 (19)	72 (64–79)	78 (66–84)	5–100	31 (20)	0.73 (0.65–0.81)	0.71 (0.59–0.92)	0.22–1.00	31 (20)	0.73 (0.66–0.79)	0.75 (0.66–0.84)	0.30–1.00
1	54 (34)	69 (64–74)	73 (60–84)	10–96	56 (36)	0.69 (0.62–0.76)	0.72 (0.59–0.88)	−0.08 to 1.00	55 (35)	0.72 (0.67–0.77)	0.73 (0.58–0.88)	0.25–1.00
2	41 (26)	69 (64–74)	70 (60–80)	25–93	39 (25)	0.61 (0.51–0.72)	0.66 (0.51–0.81)	−0.57 to 1.00	40 (25)	0.68 (0.61–0.74)	0.70 (0.55–0.84)	0.16–0.98
> 2	31 (20)	63 (56–70)	60 (50–75)	30–100	29 (19)	0.54 (0.43–0.65)	0.55 (0.30–0.73)	−0.17 to 1.00	31 (20)	0.61 (0.53–0.69)	0.64 (0.45–0.77)	0.23–0.99
BMI category (kg/m2)******												
Normal (18.5–24.9)	34 (22)	68 (62–74)	75 (60–80)	25–95	35 (23)	0.73 (0.66–0.81)	0.80 (0.66–0.87)	0.04–1.00	36 (23)	0.71 (0.64–0.77)	0.71 (0.59–0.85)	0.30–0.98
Underweight (< 18.5)	1 (1)	NA	NA	50	1 (1)	NA	NA	0.20	1 (1)	NA	NA	0.34
Overweight (25–29.9)	66 (42)	70 (65–75)	75 (60–84)	5–96	63 (41)	0.68 (0.62–0.75)	0.71 (0.58–0.86)	−0.57 to 1.00	64 (41)	0.71 (0.67–0.76)	0.74 (0.62–0.85)	0.16–1.00
Obese (> 30)	46 (29)	68 (64–73)	70 (58–80)	40–100	47 (30)	0.57 (0.49–0.66)	0.64 (0.48–0.74)	−0.20 to 1.00	47 (30)	0.65 (0.59–0.71)	0.67 (0.49–0.84)	0.22–1.00
*St. Georges Respiratory Questionnaire total score*												
Quartile 1	31 (20)	84 (80–88)	85 (78–92)	60–100	32 (21)	0.85 (0.78–0.85)	0.92 (0.81–1.00)	0.14–1.00	31 (20)	0.86 (0.80–0.92)	0.92 (0.75–0.98)	0.44–1.00
Quartile 2	36 (23)	72 (68–76)	75 (69–80)	30–90	35 (23)	0.74 (0.69–0.74)	0.72 (0.67–0.85)	0.48–1.00	36 (23)	0.76 (0.71–0.81)	0.77 (0.67–0.86)	0.35–0.95
Quartile 3	25 (16)	67 (60–73)	70 (60–80)	35–90	28 (19)	0.61 (0.53–0.61)	0.64 (0.58–0.71)	−0.17 to 0.81	27 (17)	0.64 (0.59–0.7)	0.67 (0.55–0.73)	0.23–0.88
Quartile 4	31 (20)	56 (49–63)	57 (50–68)	10–90	27 (18)	0.38 (0.23–0.38)	0.46 (0.17–0.63)	−0.57 to 1.00	31 (20)	0.54 (0.47–0.61)	0.51 (0.4–0.68)	0.16–0.93
*UCSD Shortness of Breath Questionnaire*												
Quartile 1	30 (19)	85 (81–88)	85 (80–92)	60–100	32 (21)	0.86 (0.81–0.86)	0.92 (0.74–1.00)	0.55–1.00	30 (19)	0.85 (0.8–0.91)	0.90 (0.75–0.98)	0.47–1.00
Quartile 2	32 (20)	73 (69–78)	75 (69–80)	30–90	30 (20)	0.75 (0.68–0.75)	0.76 (0.66–0.91)	0.14–1.00	32 (20)	0.78 (0.73–0.84)	0.82 (0.73–0.88)	0.35–0.98
Quartile 3	30 (19)	67 (62–72)	70 (60–75)	35–90	28 (19)	0.60 (0.51–0.60)	0.64 (0.55–0.72)	−0.17 to 0.88	32 (20)	0.65 (0.6–0.70)	0.67 (0.56–0.74)	0.23–0.90
Quartile 4	31 (20)	54 (48–61)	57 (47–65)	10–88	32 (21)	0.42 (0.30–0.42)	0.50 (0.22–0.66)	−0.57 to 1.00	31 (20)	0.54 (0.48–0.61)	0.52 (0.41–0.67)	0.16–0.93
*HADS Anxiety*												
Normal	94 (60)	73 (70–76)	75 (61–85)	30–100	96 (63)	0.72 (0.68–0.77)	0.73 (0.62–0.92)	−0.01 to 1.00	96 (61)	0.75 (0.71–0.78)	0.77 (0.67–0.89)	0.34–1.00
Mild	15 (10)	56 (47–66)	60 (50–68)	10–80	14 (9)	0.49 (0.31–0.67)	0.54 (0.48–0.70)	−0.20 to 0.86	15 (10)	0.52 (0.45–0.59)	0.56 (0.48–0.63)	0.22–0.74
Moderate	13 (8)	63 (50–75)	65 (50–75)	25–90	11 (7)	0.41 (0.21–0.61)	0.49 (0.27–0.62)	−0.17 to 0.81	13 (8)	0.41 (0.17–0.64)	0.45 (0.39–0.51)	0.23–0.72
Severe	1 (1)	b	b	b	1 (1)	b	b	b	1 (1)	b	b	b
*HADS Depression*												
Normal	103 (66)	73 (70–76)	75 (63–85)	30–100	102 (67)	0.72 (0.67–0.76)	0.73 (0.61–0.91)	−0.17 to 1.00	104 (66)	0.75 (0.71–0.78)	0.75 (0.66–0.88)	0.23–1.00
Mild	15 (10)	60 (54–67)	60 (50–68)	40–80	15 (10)	0.46 (0.31–0.61)	0.51 (0.34–0.63)	−0.20 to 0.78	16 (10)	0.52 (0.45–0.59)	0.53 (0.48–0.58)	0.22–0.82
Moderate	5 (3)	33 (10–56)	25 (25–50)	10–55	5 (3)	0.06 (−0.47 to 0.59)	0.04 (−0.08 to 0.39)	−0.57 to .51	5 (3)	0.41 (0.17–0.64)	0.42 (0.3–0.47)	0.16–0.67
Severe	–	–	–	–	–	–	–	–	–	–	–	–
**Medications**												
*Conditional recommendations for use (antifibrotics)*												
No	64 (41)	67 (63–72)	73 (57–80)	5–95	62 (40)	0.62 (0.55–0.69)	0.67 (0.50–0.80)	−0.20 to 1.00	63 (40)	0.67 (0.62–0.72)	0.70 (0.55–0.83)	0.22–0.97
Yes	92 (59)	69 (65–73)	70 (60–85)	10–100	93 (60)	0.67 (0.62–0.73)	0.70 (0.56–0.88)	−0.57 to 1.00	94 (60)	0.70 (0.66–0.74)	0.72 (0.54–0.87)	0.16–1.00
*Conditional recommendations for use (limited evidence)*												
No	78 (50)	71 (67–75)	75 (65–85)	5–100	81 (50)	0.71 (0.65–0.77)	0.72 (0.62–0.87)	−0.08 to 1.00	78 (50)	0.73 (0.69–0.77)	0.75 (0.63–0.85)	0.25–1.00
Yes	78 (50)	66 (62–69)	65 (51–80)	25–100	81 (50)	0.60 (0.53–0.67)	0.64 (0.49–0.81)	−0.57 to 1.00	79 (50)	0.65 (0.60–0.70)	0.67 (0.49–0.83)	0.16–0.99
*Strong recommendations against use*												
No	130 (83)	70 (67–73)	70 (60–84)	5–100	130 (81)	0.67 (0.63–0.71)	0.71 (0.56–0.85)	−0.57 to 1.00	130 (83)	0.72 (0.69–0.75)	0.75 (0.56–0.87)	0.16–1.00
Yes	26 (17)	61 (54–68)	60 (49–75)	25–90	30 (19)	0.33 (0.03–0.63)	0.49 (−0.01 to 0.52)	−0.20 to 1.00	27 (17)	0.55 (0.47–0.63)	0.60 (0.39–0.69)	0.22–0.9

^*^Missing values = 7; **Missing value = 1; ***Missing value = 29; ****Missing value = 2, *****Missing value = 3, ******Missing value = 9 (number of participants not reporting information for this variable)

^a^ Total number of participants varies for each instrument

^b^*n* < 5, not able to report due to ethical restriction

*n* number of participants, *HSUV* Health state utility value, *95CI*, 95 lower and upper confidence interval, *IQR* interquartile range, *min* minimum, *max* maximum, *FVC* forced vital capacity percent predicted, *GAP* Gender, age, physiology, *CPI* Composite physiological index, *NC* not classified includes participants with missing PFTs, *BMI* Body Mass Index. Conditional recommendations for use (antifibrotics) drugs include pirfenidone and nintedanib. Strong recommendations against use drugs include prednisolone, n-acetylcysteine, warfarin and azathioprine. Conditional recommendations for use (limited evidence) includes anti-reflux drugs

Univariable Tobit models (Table S3) indicated that the disease severity measures (PFTs), > 2 comorbidities, employment status, and medications in the categories “strong recommendations against use” and “conditional recommendations for use” were statistically significant predictors of HSUVs for both instruments, which was consistent with the descriptive analysis. The AQoL-8D unlike the EQ-5D-5L showed statistically significant associations between all age groups and HSUVs (reference age grou*p* ≤ 65 years) and the EQ-5D-5L demonstrated a statistically significant association with BMI and HSUVs, which was not observed with the AQoL-8D. For the multivariable models (Table S4–S6), our results demonstrated similar significant associations for both instruments for disease severity, persons with > 2 comorbidities, and employment status. The magnitude of the effect for the most part was larger with the EQ-5D-5L. The AQoL-8D however demonstrated additional statistically significant associations with age groups and medications in the category “strong recommendations against use” (Table 6).

### Test performance

#### Internal consistency

Cronbach alpha scores (Table S7) for the EQ-5D-5L and AQoL-8D were 0.83 (95%CI, 0.79–0.87) and 0.95 (95%CI, 0.94–0.96), respectively. Closer evaluation of the AQoL-8D revealed Cronbach alpha scores between 0.80 and 0.90 for all dimensions except for coping (0.59) and senses (0.22).

#### Construct validity

The AQoL-8D was very strongly correlated with EQ-5D-5L (0.80). The PSD (0.79) was more strongly associated with the EQ-5D-5L utility than the MSD (0.74). The EQ-VAS was strongly associated with both the AQoL-8D and the EQ-5D-5L, 0.66 and 0.63, respectively. The SOBQ and SGRQ were more strongly associated with the EQ-5D-5L and the HADS and SGRQ impact with the AQoL-8D. More details are provided in Table S8.

Both instruments were able to detect statistically significant differences in HSUVs between clinical variables. The effect size between groups was larger for the EQ-5D-5L. The AUC was larger for the EQ-5D-5L indicating a higher sensitivity to differences in HSUVs between groups and the RE reflected that the EQ-5D-5L was more efficient in detecting differences between groups than the AQoL-8D. Full details are provided in Table S9.

## Discussion

Given the importance of health economic evaluations in health financing decision-making especially with the expanding landscape of treatments for IPF, the selection of a preference-based PROM for research is a critical undertaking. Consequently, our study sought to directly compare the AQoL-8D and EQ-5D-5L for measuring HRQoL in persons with IPF in Australia. There was reasonable agreement between the two instruments for measuring HRQoL, however, there were some fundamental differences. One of these key differences was the enhanced sensitivity of the AQoL-8D compared to the EQ-5D-5L to measure the psychosocial aspects of HRQoL. This was further confirmed when the instruments were compared to other HRQoL measures. The EQ-5D-5L was highly correlated with the SOBQ and the Activity component of the SGRQ while the AQoL-8D was more associated with the Impact domain of the SGRQ and the HADS. In contrast to the AQoL-8D, the EQ-5D-5L had a greater divergent sensitivity and efficacy in relation to assessing HRQoL between clinical groupings.

Our study demonstrated that in this cohort, the practicality of both instruments was similar, noting that completion rates were sufficient for estimation of HSUVs, and extreme responses, were comparable for both instruments. Closer examination of fully completed questionnaires demonstrated an 11% difference in completion rates favouring the EQ-5D-5L, despite the AQoL-8D being administered first. This was consistent with published literature in a similarly aged population [39], and was expected given the difference in length in the questionnaires: 35 items with completion time of 5.5 min for the AQoL-8D and 5 items with completion time of 1 min for the EQ-5D-5L [5].

There was reasonable agreement between the EQ-5D-5L and AQoL-8D. First, the mean and median HSUVs for the AQoL-8D and EQ-5D-5L were similar, with the differences between the means and medians being 0.04 and 0.02 respectively. While there is limited evidence on the minimally important difference (MID) for the AQoL-8D or EQ-5D-5L for IPF, these differences fall within the reported MIDs in published literature for these two instruments, for the general Australian population (0.06 (0.03–0.08)) [40] and for a Canadian IPF cohort (0.01–0.05) [41], signifying that there was consistency in the health status between the two measures. The Bland–Altman plot, ICC and regression analysis provided further evidence to support the agreement between the AQoL-8D and EQ-5D-5L. While our study demonstrated reasonable agreement, previous studies that have evaluated the two instruments have shown larger discrepancies and lower HSUVs with AQoL-8D compared to the EQ-5D-5L [34, 39, 41]. We attribute this difference to disease or population specific characteristics which may be more focussed on psychosocial deficits to which the AQoL-8D is more responsive [42, 43], whereas for IPF the deficits related to the symptoms are predominantly physical [4], to which the EQ-5D-5L is predominantly responsive [43, 44]. This was further substantiated when we assessed the convergent validity, more specifically the association between the AQoL-8D and EQ-5D-5L and the disease specific or symptom related measures of HRQoL, where we noted strong associations between the EQ-5D-5L and the activity component of the SGRQ and the SOBQ while the AQoL-8D was strongly associated with the impact domain of the SGRQ and the HADS questionnaire.

Notwithstanding the similarities, there were notable differences which provided insight into the suitability of the AQoL-8D and EQ-5D-5L in an IPF cohort. In the first instance, the EQ-5D-5L demonstrated a wider range of HSUVs (−0.57 to 1.00 vs 0.16–1.00) and also demonstrated a larger proportion of persons with floor (4%) and ceiling effects (13%). This suggests that the EQ-5D-5L may not be a sufficiently sensitive measure for mild disease, but it may be more responsive to severe disease compared to the AQoL-8D. This is possibly as a result of the high symptom burden which is physically debilitating in persons with severe IPF as compared to milder disease. Conversely, in this cohort, the AQoL-8D is evidently a more robust measure for milder disease, demonstrating a wider range of HSUVs between 0.68 and 1.00 for this subgroup of patients who scored full health (1.00) with the EQ-5D-5L, noting that most of the deficit was attributed to the AQoL-8D MSD (psychosocial). While there is no comparison study for the AQoL-8D in an IPF cohort, recent research with the EQ-5D-5L has shown similar ceiling effects in patients with milder disease [41], corroborating our findings.

An important characteristic of a PROM is the ability to differentiate between known groups that are clinically different. To assess this, we focussed on clinically relevant variables. Our results demonstrated that both instruments were able to detect HSUV differences between groups in the variables studied. The EQ-5D-5L demonstrated a larger ES, higher sensitivity (AUC) and efficiency (RE) than the AQoL-8D, for clinical groups based on lung function testing. This was also seen in our regression analysis that demonstrated larger effect sizes with the EQ-5D-5L than with the AQoL-8D for GAP, FVC% and CPI. Of note however is the magnitude of the AUC for both the EQ-5D-5L and the AQoL-8D, both less than 0.75, indicative of a lower than optimal discriminatory power [45]. While this is not ideal, it is expected as generic instruments may not be sensitive enough to detect minimal changes related to disease specific or clinical parameters and is the reason for the recommendation to use these alongside disease specific instruments in IPF cohorts [4, 41]. Conversely, the AQoL-8D demonstrated a higher sensitivity and efficiency to differentiate clinical classification groupings with the HADS and the SGRQ, consistent with its responsiveness to the psychosocial aspects of HRQoL.

While there are no established standards for assessing HRQoL in IPF [4], and more specifically as it relates to preference-based instruments, the instrument selection process should be guided by its sensitivity to the unique characteristics of IPF patients and the specific changes expected by the interventions being evaluated. Notwithstanding the fact that there is no perfect instrument [5, 8], instruments with low sensitivity to changes in health states attributed to an intervention, or not suited to the specific population, may potentially introduce unwanted bias in the decision-making process [5, 8]. The EQ-5D-5L may potentially be more suited to our IPF cohort, primarily because of the evidence supporting its practicality, the wide observed range of HSUVs and its superior divergent validity, the latter of utmost importance when evaluating new treatments or interventions. While we acknowledge that the EQ-5D-5L may not fully capture the psychosocial aspects of HRQoL, our results demonstrated that the mean and median HSUVs from both instruments were quite similar and within MIDs, suggesting that this deficit may not be the primary influencer of the HSUVs, especially noting that HSUVs were higher with the AQoL-8D instrument. We do not, however, disregard the advantages of the AQoL-8D and recommend that they be used together whenever possible, especially in cohorts with milder disease.

This study has generated the first HSUVs for an Australian cohort of persons living with IPF, and the first to undertake a comparison of the AQoL-8D and EQ-5D-5L in a cohort of persons with IPF. This will be useful in future economic evaluations and adds to the limited evidence on preference-based instruments in the field of IPF. There are however some limitations, firstly the small cohort size. As IPF is a rare disease, this is consistent with other research [4]. Recent research estimates approximately 11,000 persons living with IPF in Australia [46], suggesting a 7–8% margin of error at a 95% confidence level with our cohort. In addition to the sample size, our cohort may not fully represent the Australian IPF population as both the AIPFR and survey were opt-in. This may mean that persons with more severe disease and older persons may be disproportionately represented in our cohort, and this may possibly underestimate the effect of IPF on HSUVs. However, we conducted a comparison in an earlier study and the results were analogous to results from other countries [11].

A second limitation is the cross-sectional nature of the study. This firstly limits our assessment of the construct validity as it relates to the sensitivity of the instruments to detect changes over time, which would be relevant to the context of economic evaluation. This will be one of the subjects of our continued research. Additionally, we used cross-sectional data for lung function and disease specific HRQoL instruments that were within 12 months of the survey completion. While this may be acceptable in most cases, progression of the disease can be quite varied, and this timeline may not be ideal in the case of rapid progressors [47].

A further limitation of this study is that we did not compare the instruments based on content and structural validity while this is an essential part of validating an instrument for use in a specific population, this was not the aim of this study. A comprehensive validation of both instruments will be the focus of our future work. Our analysis however demonstrated that the behaviour of the two instruments in this cohort was in line with previous evaluations of content validity which demonstrated a predisposition of the content of the EQ-5D to measure physical deficits/attributes of HRQoL and the AQoL to measure psychosocial deficits/attributes [5–9, 43, 44].

Finally, the assumptions used in the estimation of the EQ-5D-5L HSUVs, however the sensitivity analysis conducted in our previous study demonstrated that the values generated from the cross walk method [14] and UK value set were similar to the estimates generated from the Australian value set [4]. Despite this, we will update the analysis once a published value set is available from EuroQol, although we do not believe this will change our outcomes.

## Conclusion

In selecting a MAUI for economic evaluation in a specific disease area, it is important to understand their descriptive systems and their innate characteristics as it relates to the disease being evaluated. Our study, the first of its kind, aimed to assess this for the AQoL-8D and EQ-5D-5L. Our findings suggest the EQ-5D-5L is the preferred instrument in for use in IPF based on the criteria evaluated, given its inherent sensitivity in measuring physical attributes related to HRQoL, and the debilitating physical effects of the symptoms of IPF.

## Supplementary Information

Below is the link to the electronic supplementary material.

Supplementary file1 (PDF 312 KB)

## Data Availability

The datasets generated and code used for the analysis are available from the corresponding author upon reasonable request.
