# Supplementary material for: Assessment of health-related quality of life in Australian patients with idiopathic pulmonary fibrosis: a comparison of the EQ-5D-5L and the AQoL-8D
Source: Qual Life Res. 2022 Aug 4;32(2):473–93. doi: 10.1007/s11136-022-03205-z (PMC9911478; doi:10.1007/s11136-022-03205-z)
Supplement: Supplementary file 1 — Supplementary file1 (PDF 312 KB) [file 11136_2022_3205_MOESM1_ESM.pdf]

Assessment of Health-Related Quality of Life in Australian patients with Idiopathic Pulmonary Fibrosis: A comparison of the EQ-5D-5L and the AQoL-8D

Ingrid A. Cox[1,2], Julie Campbell[1], Barbara de Graaff[1,2], Petr Otahal[1], Tamera J. Corte [2,3,4], Yuben Moodley[2,7,8,9], Peter Hopkins[2,10,11], Sacha Macansh[12], *E. Haydn Walters[1,2], *Andrew J. Palmer[1,2].

[1] Menzies Institute for Medical Research, University of Tasmania, Tasmania, Australia.

[2] NHMRC Centre of Research Excellence for Pulmonary Fibrosis, Australia.

[3]Central Clinical School, The University of Sydney, Camperdown, Australia.

[4]Department of Respiratory and Sleep Medicine, Royal Prince Alfred Hospital, Camperdown, Australia.

[7]Faculty of Health and Medical Sciences, The University of Western Australia, Perth, Australia

[8]Institute of Respiratory Health, The University of Western Australia, Perth, Australia

[9]Department of Respiratory Medicine, Fiona Stanley Hospital, Murdoch, Australia

[10]Queensland Centre for Pulmonary Transplantation and Vascular Disease, The Prince Charles Hospital, Chermside, Australia

[11]Faculty of Medicine, University of Queensland, Queensland, Australia.

[12]Australian Idiopathic Pulmonary Fibrosis Registry, Lung Foundation of Australia, New South Wales, Australia

*Joint senior authors

<u>Correspondence to:</u>

Professor Andrew Palmer

Menzies Institute for Medical Research, University of Tasmania,

17 Liverpool Street, Hobart, Tasmania, Australia.

Email: andrew.palmer@utas.edu.au

Phone: +61 362267700

Supplementary material

Disease severity measures

GAP staging [23] uses the four parameters gender, age, FVC% and the diffusing capacity of the lungs for carbon monoxide percent predicted (DLco%). Patients are then classified into stages based on the summation of scores (index) which can range from 0-8 namely, Stage I (GAP index: 0−3), Stage II (GAP index: 4−5) and Stage III (GAP index: 6−8), with Stage I being the least severe. The CPI is calculated using a predetermined formula [24,26] based on the FVC%, DLco% and the forced expiratory volume as a percent predicted (FEV%). Higher CPI scores correspond to more severe disease and cut-off points of CPI≤40 has been considered mild disease and CPI>40 as moderate to severe disease [26]. For the FVC%, higher FVC % scores correspond to milder disease. Cut-off points of FVC%>75% have been considered as mild disease, FVC% between 50-75% as moderate disease, and FVC%<50% as severe disease [22-23, 24-26]. For the CPI and the FVC% we used quintiles since there are varying perspectives on cut-off points for disease severity categorisation.

Recruitment methods

Australian IPF Registry

The Australian Idiopathic Pulmonary Fibrosis Registry(AIPFR) was established in 2012. It is a multi-centre, prospective, observational registry of incident and prevalent IPF patients from every State and Territory in Australia. The registry is an opt-in registry and, collects serial data and clinical investigations from participants and physicians and operates in tandem with a linked biobank. Recruitment is not limited to tertiary institutions and all pulmonologists operating in Australia can refer patients with a diagnosis of IPF to the Registry. Once participants have consented to the Registry, the IPF diagnosis is re-evaluated by a multidisciplinary panel and the diagnosis is classified based on American Thoracic Society/European Respiratory Society/Japanese Respiratory Society/Latin American Thoracic Association (ATS/ERS/JRS/ALAT) guidelines. Baseline data are also collected via predesigned proformas from the participants and clinicians, and thenceforth every 6 months. The 6 monthly follow up data collected includes participant and physician proformas, medical progress, examination findings as well as results of investigations performed. All data is remotely entered into a central database managed by the Lung Foundation of Australia.

Study participants

Inclusion criteria for this study were: a current diagnosis of IPF, enrolment in the Australian IPF Registry, aged 18 years and older and ability to provide informed consent. All participants were invited to consent and once consented were provided with a questionnaire for completion which collected socio-demographic information, information on comorbidities, treatment and incorporated the EQ-5D-5L and AQoL-8D instrument.

Figure S1 Flow chart illustrating participant selection

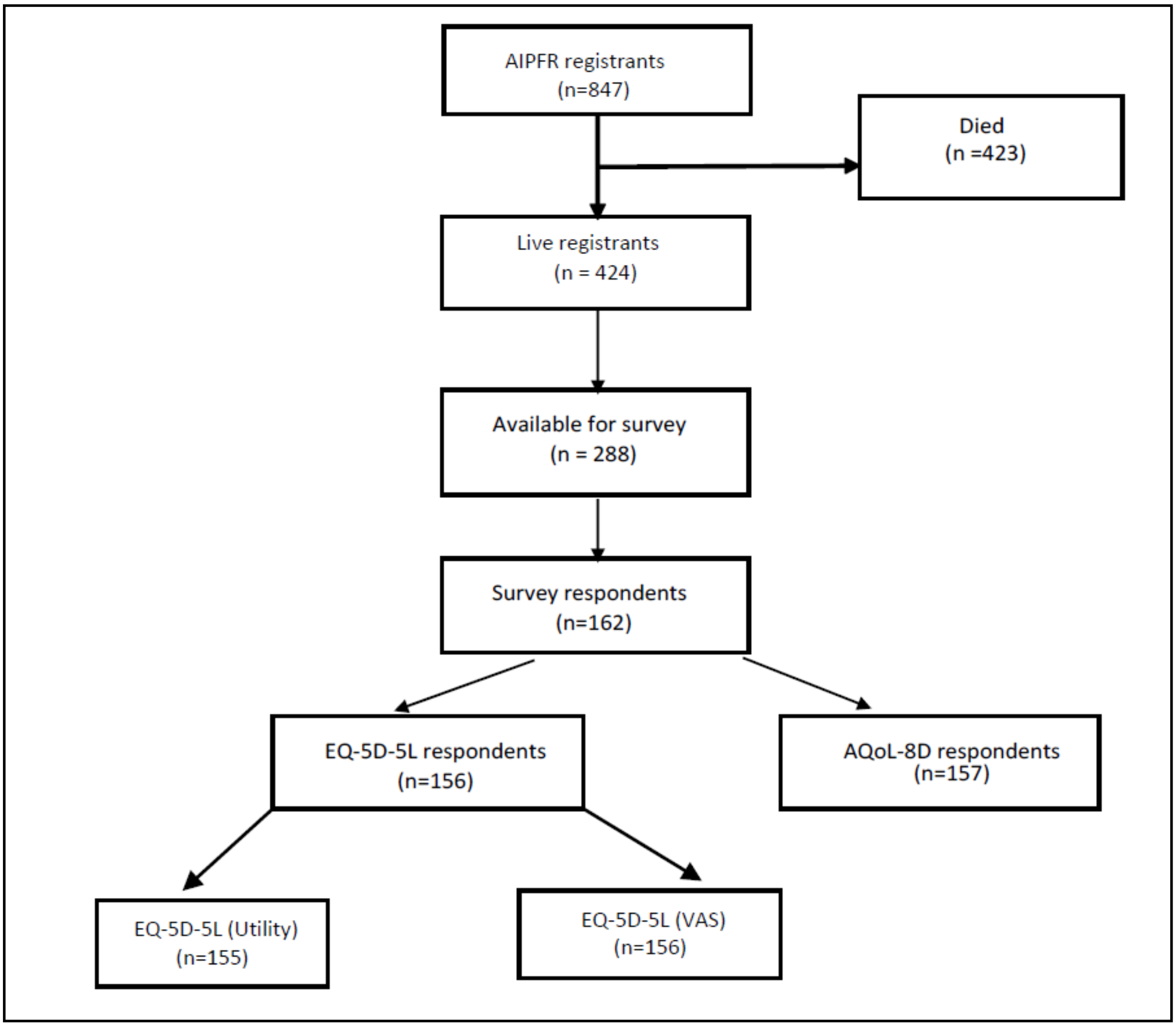

AIPFR, Australian IPF Registry; n, number of participants; VAS, visual analogue scale.

Table S1 Comparison of non-responders and participants in the survey

	**Non-responders (n=126)**	**Participants (n=162)**	**p value**	**Comparator survey results (n=129)**	**No comparator survey results (n=33)**	**p value**	**PFTs (n=105)**	**No PFTs (n=57)**	**p value**
**Age (years)**			0.20			0.16			0.57
Mean (SD)	75.2 (9.1)	73.9 (7.5)		74.3(7.74)	72.0(6.87)		73.6(7.44)	74.3(7.95)	
**Age group, years, n (%)**			**0.00**			0.42			0.61
<65	18 (14.3)	18 (11.1)		13(10.1)	6(18.2)		13(12.4)	6(10.5)	
65 75	38 (30.2)	83 (51.2)		65(50.4)	17(51.5)		51(48.6)	31(54.4)	
75 85	57 (45.2)	48 (29.6)		39(30.2)	9(27.3)		34(32.4)	14(24.6)	
85+	13 (10.3)	13 (8.0)		12(9.3)	1(3.0)		7(6.7)	6(10.5)	
**Sex, n (%)**			0.91			1.00			0.65
Male	81 (64.3)	102 (63.0)		79(61.2)	20(60.6)		6 (62.9)	3 (57.9)	
Female	45 (35.7)	60 (37.0)		50(38.8)	13(39.4)		39(37.1)	24(42.1)	
**Ethnicity, n (%)**			0.34			1.00			0.25
Caucasian	111 (88.1)	145 (89.5)		17(90.7)	28(84.8)		93(8 .6)	52(91.2)	
Other	3 (2.4)	9 (5.6)		7(5.4)	2(6.1)		8(7.6)	1(1.8)	
**Comorbidities, n (%)**			**<0.001**			0.48			0.81
None	13 (10.3)	33 (20.4)		26(20.2)	7(21.2)		20(19.0)	13(2 .8)	
One	30 (23.8)	56 (34.6)		42(32.6)	14(42.4)		36(34.3)	20(35.1)	
Two	32 (25.4)	41 (25.3)		36(27.9)	5(15.2)		29(27.6)	12(21.1)	
More than two	49 (38.9)	32 (19.8)		25(19.4)	7(21.2)		20(19.0)	12(21.1)	
**BMI (kg/m2)**	-	-	-			0.25			**0.04**
Mean (SD)	-	-	-	28.1(4.57)	28.5(5.70)		27.6(4.30)	29.5(5.58)	
**Recommended Drugs, n (%)**	-	-	-			**0.02**			**0.01**
No	-	-	-	46(35.7)	20(60.6)		34(32.4)	32(56.1)	
Yes	-	-	-	83(64.3)	13(39.4)		71(67.6)	25(43.9)	
**Limited Evidence Drugs, n (%)**	-	-	-			0.44			1.00
No	-	-	-	67(51.9)	14(42.4)		53(50.5)	28(49.1)	
Yes	-	-	-	62(48.1)	19(57.6)		52(49.5)	29(50.9)	
**Not Recommended Drugs, n (%)**	-	-	-			**0.002**			**0.01**
No	-	-	-	114(88.4)	21(63.6)		94(89.5)	41(71.9)	
Yes	-	-	-	15(11.6)	12(36.4)		1 (10.5)	16(28.1)	

n, number of participants; PFT, lung function tests; SD, standard deviation; BMI, body mass index; Recommended drugs include pirfenidone and nintedanib. Not recommended drugs include N-acetylcysteine, warfarin and azathioprine. Limited evidence drugs include prednisolone and anti-reflux drugs. Comparator surveys include St George's Respiratory Questionnaire, University of California San Diego Shortness of Breath Questionnaire and the Hospital Anxiety and Depression Scale. p value for appropriate test comparing disease classification groups (Chi-squared test or one-way ANOVA test). Bolded results represents statistically significant results ($p<0.05$).

Table S2 Response rates for AQoL-8D and EQ-5D-5L

	AQOL-8D								EQ-5D-5L				
**Level**	**Mental health**	**Coping**	**Happiness**	**Relationships**	**Self-worth**	**Pain**	**Independent living**	**Senses**	**Mobility**	**Self-care**	**Usual activities**	**Pain/ Discomfort**	**Anxiety/ Depression**
**1**	32.9	18.5	17.7	42.9	32.7	32.7	34.0	50.3	34.6	67.3	27.2	27.2	55.6
**2**	32.5	51.0	52.9	31.1	35.8	43.8	29.0	26.9	27.8	19.1	38.9	40.1	30.9
**3**	26.5	18.9	22.4	18.9	23.9	15.1	30.9	19.1	25.9	9.9	22.2	26.5	10.5
**4**	6.0	8.0	5.1	4.7	6.0	12.3	4.3	2.8	9.9	3.1	7.4	2.5	0.6
**5**	1.7	2.7	1.1	0.9	1.2	5.9	0.6	1.2	1.2	0.6	3.7	0.6	1.2
**6**	0.6	NA	NA	0.0	NA	NA	4.9	0.0	NA	NA	NA	NA	NA

Table S3 Univariable regression results for AQoL-8D, EQ-5D-5L and EQ-VAS based on participant characteristics

	EQ-5D-5L utility	AQoL-8D utility
**Age group**		
<65	ref	ref
65-75	0.11	**0.14**
75-85	0.08	**0.14**
>85	0.16	**0.19**
**Gender**		
Male	ref	ref
Female	-0.08	-0.05
**Race**		
Caucasian	ref	ref
Other	0.07	-0.05
**Jurisdiction**		
NSW	ref	ref
VIC	0.03	0.01
QLD	-0.11	-0.10
SA	-0.09	-0.05
TAS	-0.10	-0.05
WA	0.02	0.00
ACT	0.14	0.16
NT	-0.08	-0.16
**Remoteness area**		
Major city	ref	ref
Inner regional	-0.06	-0.05
Outer regional	-0.10	-0.08
Remote	-0.08	-0.16
**Marital Status,**		
Married/De facto/Partner	ref	ref
Divorced/Widowed/Separated/Single	0.00	-0.02
**Employment**		
Full time/Part time/Unpaid work	ref	ref
Retired	**-0.17**	**-0.17**
Unemployed	**-0.28**	**-0.29**
**Income($AUD)**		
<400/week	ref	ref
400-799/week	0.04	0.03
800-1249/week	0.00	0.02
>1250/week	0.13	0.07
**Comorbidities**		
0	ref	ref
1	-0.04	-0.01
2	-0.12	-0.05
>2	**-0.19**	**-0.12**
**BMI kg/m2**	**-0.01**	0.00
**FVC% (10-unit increase)**	**0.03**	**0.02**
**GAP index (1 unit increase)**	**-0.04**	**-0.02**
**CPI (10-unit increase)**	**-0.05**	**-0.03**
**Medications**		
Conditional recommendations for use (antifibrotics)	0.06	0.03
Conditional recommendations for use (limited evidence)	**-0.09**	**-0.08**
Strong recommendations against use	**-0.13**	**0.17**

BMI, body mass index; FVC, forced vital capacity percent predicted; GAP, Gender, Age, Physiology; CPI, Composite Physiological Index. NSW, New South Wales; VIC, Victoria; SA, South Australia; QLD, Queensland; TAS, Tasmania; WA, Western Australia; ACT, Australian Capital Territory; NT, Northern Territory. Recommended drugs include pirfenidone and nintedanib. Not recommended drugs include N-acetylcysteine, warfarin and azathioprine. Limited evidence drugs include prednisolone and anti-reflux drugs. Bolded results represent statistically significant results (p<0.05).

Table S4 Multivariable regression analysis results for FVC%

	EQ-5D-5L utility	AQoL-8D utility
**Age group**		
<65	ref	ref
65-75	0.05	0.08
75-85	0.01	0.09
>85	0.07	0.13
**Employment,**		
Full time/Part time/Unpaid work	ref	ref
Retired	**-0.20**	**-0.20**
Unemployed	**-0.30**	**-0.26**
**Comorbidities**		
0	ref	ref
1	0.00	0.00
2	-0.07	-0.03
>2	**-0.17**	**-0.11**
**BMI kg/m2**	0.00	0.00
**FVC% (10-unit increase)**	**0.03**	**0.01**
**Medications**		
Conditional recommendations for use (antifibrotics)	0.03	0.00
Conditional recommendations for use (limited evidence)	-0.03	-0.03
Strong recommendations against use	-0.03	**-0.11**

BMI, body mass index; FVC, forced vital capacity percent predicted; Recommended drugs include pirfenidone and nintedanib. Not recommended drugs include N-acetylcysteine, warfarin, and azathioprine. Limited evidence drugs include prednisolone and anti-reflux drugs. Bolded results represent statistically significant results ($p<0.05$).

Table S5 Multivariable regression analysis results for GAP index

	EQ-5D-5L utility	AQoL-8D utility
**Age group**		
<65		
65-75	0.08	0.10
75-85	0.07	**0.13**
>85	0.14	**0.18**
**Employment,**		
Full time/Part time/Unpaid work	ref	ref
Retired	**-0.20**	**-0.20**
Unemployed	**-0.30**	**-0.22**
**Comorbidities**		
0	ref	ref
1	-0.03	-0.03
2	-0.08	-0.04
>2	**-0.20**	**-0.13**
**BMI kg/m2**	-0.01	
**GAP(1 unit increase)**	**-0.06**	**-0.03**
**Medications**		
Conditional recommendations for use (antifibrotics)	0.06	0.02
Conditional recommendations for use (limited evidence)	-0.04	-0.04
Strong recommendations against use	-0.06	**-0.12**

BMI, body mass index; GAP, Gender, Age, Physiology; Recommended drugs include pirfenidone and nintedanib. Not recommended drugs include N-acetylcysteine, warfarin, and azathioprine. Limited evidence drugs include prednisolone and anti-reflux drugs. Bolded results represent statistically significant results ($p<0.05$).

Table S5 Multivariable regression analysis results for CPI

	EQ-5D-5L utility	AQoL-8D utility
**Age group**		
<65	ref	ref
65-75	0.04	0.06
75-85	0.03	0.10
>85	0.11	**0.15**
**Employment,**		
Full time/Part time/Unpaid work	ref	ref
Retired	**-0.20**	**-0.19**
Unemployed	**-0.29**	**-0.22**
**Comorbidities**		
0	ref	ref
1	-0.02	-0.03
2	-0.08	-0.04
>2	-0.21	**-0.13**
**BMI kg/m2**	-0.01	0.00
**CPI (10-unit increase)**	**-0.06**	**-0.03**
**Drugs,**		
Conditional recommendations for use (antifibrotics)	0.07	0.03
Conditional recommendations for use (limited evidence)	-0.03	-0.03
Strong recommendations against use	-0.03	**-0.11**

BMI, body mass index; CPI, Composite Physiological Index. Recommended drugs include pirfenidone and nintedanib. Not recommended drugs include N-acetylcysteine, warfarin, and azathioprine. Limited evidence drugs include prednisolone and anti-reflux drugs. Bolded results represent statistically significant results ($p<0.05$).

Table S7 Internal consistency analysis (Cronbach α scores)

	Number of items	Cronbach alpha (95%CI)
**EQ-5D-5L**	5	0.83(0.79-0.87)
**AQoL-8D**	35	0.95(0.94-0.96)
**AQoL-8D Psychosocial health**		
Mental health	8	0.85(0.82-0.88)
Coping	3	0.59(0.48-0.70)
Happiness	4	0.86(0.83-0.89)
Relationships	7	0.83(0.80-0.87)
Self-worth	3	0.80(0.75-0.85)
**AQoL-8D Physical Health**		
Pain	3	0.82(0.79-0.86)
Independent living	4	0.90(0.87-0.92)
Senses	3	0.22(0.02-0.42)

Table S8 Convergent validity analysis with the Spearman's rank order correlation test

	Utility	EQ-VAS	MSD	PSD	AQoL-8D Utility	IL	HAP	MH	Cop	Rel	SW	Pain	Senses	SOBQ	SYM	ACT	IMP	TOT	ANX	DEP
Utility	**1.00**																			
EQ-VAS	0.63	**1.00**																		
MSD	0.74	0.63	**1.00**																	
PSD	0.79	0.63	0.69	**1.00**																
AQoL-8 Utility	0.80	0.66	0.97	0.82	**1.00**															
IL	0.76	0.72	0.64	0.79	0.71	**1.00**														
HAP	0.58	0.63	0.83	0.50	0.77	0.52	**1.00**													
MH	0.60	0.43	0.84	0.52	0.82	0.36	0.67	**1.00**												
Cop	0.60	0.59	0.77	0.59	0.77	0.56	0.58	0.55	**1.00**											
Rel	0.64	0.58	0.83	0.63	0.82	0.64	0.64	0.55	0.60	**1.00**										
SW	0.69	0.57	0.89	0.62	0.87	0.62	0.65	0.74	0.65	0.67	**1.00**									
Pain	0.61	0.39	0.5	0.84	0.63	0.44	0.34	0.47	0.41	0.41	0.43	**1.00**								
Senses	0.33	0.29	0.44	0.49	0.51	0.26	0.34	0.36	0.31	0.41	0.33	0.26	**1.00**							
SOBQ	-0.61	-0.55	-0.5	-0.58	-0.55	-0.67	-0.36	-0.33	-0.42	-0.53	-0.48	-0.36	-0.21	**1.00**						
SYM	-0.46	-0.32	-0.37	-0.44	-0.42	-0.44	-0.32	-0.29	-0.26	-0.35	-0.30	-0.33	-0.24	0.60	**1.00**					
ACT	-0.61	-0.54	-0.49	-0.61	-0.56	-0.71	-0.38	-0.33	-0.40	-0.51	-0.51	-0.40	-0.18	0.82	0.61	**1.00**				
IMP	-0.59	-0.50	-0.59	-0.57	-0.62	-0.58	-0.51	-0.46	-0.46	-0.53	-0.50	-0.42	-0.27	0.78	0.71	0.76	**1.00**			
TOT	-0.62	-0.52	-0.56	-0.61	-0.61	-0.64	-0.47	-0.42	-0.43	-0.54	-0.50	-0.43	-0.26	0.84	0.80	0.89	0.95	**1.00**		
ANX	-0.53	-0.35	-0.66	-0.51	-0.67	-0.43	-0.46	-0.65	-0.51	-0.53	-0.61	-0.42	-0.27	0.53	0.45	0.47	0.58	0.57	**1.00**	
DEP	-0.53	-0.53	-0.65	-0.57	-0.67	-0.55	-0.6	-0.50	-0.48	-0.59	-0.53	-0.40	-0.31	0.66	0.45	0.56	0.65	0.63	0.63	**1.00**

MSD, AQoL-8D mental health super-dimension; PSD, AQoL-8D physical health super-dimension; IL, AQoL-8D independent living; HAP, AQoL-8D happiness; MH, AQoL-8D mental health; Cop, AQoL-8D coping; Rel, AQoL-8D relationships; SW, AQoL-8D self-worth; SOBQ, University of California, San Diego Shortness of Breath Questionnaire; SYM, St. George's Respiratory Questionnaire symptoms; ACT, St. George's Respiratory Questionnaire activity; IMP, St. George's Respiratory Questionnaire impact; TOT, St. George's Respiratory Questionnaire total; ANX, Hospital Anxiety and Depression Scale anxiety domain; DEP, Hospital Anxiety and Depression Scale depression domain.

Table S9 Divergent validity for EQ-5D-5L and AQoL-8D

	EQ-5D-5L	p value	ES	AUC	Aqol-8D	p value	ES	AUC	RE
**FVC quintiles**		**0.02**	0.06	0.73		**0.02**	0.06	0.63	0.94
Q1	0.48(0.26-0.48)				0.63(0.52-0.74)				
Q2	0.67(0.58-0.67)				0.70(0.62-0.78)				
Q3	0.75(0.67-0.75)				0.76(0.67-0.85)				
Q4	0.78(0.71-0.78)				0.78(0.72-0.85)				
Q5	0.71(0.57-0.71)				0.72(0.61-0.83)				
**GAP stage**		**0.001**	0.08	0.67		**0.004**	0.07	0.65	0.76
I	0.70(0.61-0.79)				0.71(0.64-0.78)				
II	0.72(0.64-0.79)				0.76(0.71-0.81)				
III	0.37(0.04-0.70)				0.54(0.37-0.7)				
**CPI Quintiles**		**0.02**	0.06	0.65		**0.04**	0.04	0.59	0.74
Q1	0.78(0.7-0.85)				0.76(0.67-0.85)				
Q2	0.75(0.59-0.9)				0.76(0.64-0.88)				
Q3	0.67(0.52-0.83)				0.72(0.63-0.82)				
Q4	0.66(0.53-0.78)				0.72(0.63-0.81)				
Q5	0.54(0.38-0.71)				0.64(0.55-0.72)				
**SGRQ total quartiles**		<**0.001**	0.28	0.82		**<0.001**	0.30	0.76	1.15
Q1	0.85(0.78-0.85)				0.86(0.80-0.92)				
Q2	0.74(0.69-0.74)				0.76(0.71-0.81)				
Q3	0.61(0.53-0.61)				0.64(0.59-0.7)				
Q4	0.38(0.23-0.38)				0.54(0.47-0.61)				
**SOBQ Total quartiles**		<**0.001**	0.30	0.85		**<0.001**	0.30	0.76	0.99
Q1	0.86(0.81-0.86)				0.85(0.8-0.91)				
Q2	0.75(0.68-0.75)				0.78(0.73-0.84)				
Q3	0.60(0.51-0.60)				0.65(0.6-0.70)				
Q4	0.42(0.30-0.42)				0.54(0.48-0.61)				
**HADS Anxiety**		**<0.001**	0.13	0.72		**<0.001**	0.28	0.80	3.82
Normal	0.72(0.67-0.76)				0.75(0.71-0.78)				
Mild	0.46(0.31-0.61)				0.52(0.45-0.59)				
Moderate	0.06(-0.47-0.59)				0.41(0.17-0.64)				
Severe	-0.57				0.16				
**HADS Depression**		**<0.001**	0.15	0.66		**<0.001**	0.18	0.72	1.45
Normal	0.72(0.67-0.76)				0.75(0.71-0.78)				
Mild	0.46(0.31-0.61)				0.52(0.45-0.59)				
Moderate	0.06(-0.47-0.59)				0.41(0.17-0.64)				
Severe	-				-				

AUC, area under the receiver operating characteristic curve; ES, effect size; RE, relative efficiency; FVC, forced vital capacity percent predicted; GAP, Gender, Age, Physiology; CPI, Composite Physiological Index SOBQ, University of California, San Diego Shortness of Breath Questionnaire; SGRQ, St. George's Respiratory Questionnaire; HADS, Hospital Anxiety and Depression Scale. Bolded results represent statistically significant results (p<0.05).
